# Olfactory training: perspective from people who were disturbed by their smell problems

**DOI:** 10.1007/s00405-024-08911-7

**Published:** 2024-08-23

**Authors:** Zetian Li, Robert Pellegrino, Christine Kelly, Thomas Hummel

**Affiliations:** 1 Department of Otorhinolaryngology, Faculty of Medicine Carl Gustav Carus, Smell & Taste Clinic, Technische Universität Dresden, Fetscherstraße 74, 01307 Dresden, Germany; 2 Monell Chemical Senses Center, Philadelphia, PA USA; 3 14 London Road, Andover, Hampshire UK

**Keywords:** Adherence, Olfactory training, Quality of life, Smell

## Abstract

**Purpose:**

Olfactory training (OT) is an effective and affordable option in the treatment of olfactory dysfunction. Despite significant progress in the field in recent years, some factors influencing OT participation remain unclear.

**Methods:**

Based on an anonymous online survey orchestrated by AbScent.org the present study enrolled 450 participants and divided them into OT (*n* = 161) and No OT (*n* = 289) groups based on their OT participation. Participants also provided information on demographics, medical history, quality of life, OT duration for those who engaged in OT, and the reasons for non-participation in OT among those who did not.

**Results:**

Patients who had greater loss of quality of life participated more in OT. Similarly, more participation was observed in patients who noticed an improvement in their ability to smell. Notably, most of the sample engaged in OT trained less than four weeks (73%). In the No OT group, the primary barrier to OT participation was the unawareness of OT treatment (37%) and these barriers differed by age, where older people expressed interest but were unaware of OT treatment, while younger individuals exhibited more cautiousness about its effectiveness.

**Conclusion:**

Lower quality of life drives active OT participation. Limited training periods and unawareness of OT serve as potential barriers to olfactory recovery. Clinicians should actively promote the background of OT and underscore the significance of adhering to the “prescribed” training regimen.

**Supplementary Information:**

The online version contains supplementary material available at 10.1007/s00405-024-08911-7.

## Introduction

Olfaction links closely to environmental safety [1], quality of life [2], social relationships [3], and emotion [4]. However, individuals with an impaired sense of smell are often unaware of their olfactory problems [5, 6].

With the pandemic COVID-19, olfaction has received unprecedented attention due to the related potentially life-threatening symptoms of the infectious disease [7]. Olfactory training (OT), an intervention that requires individuals to sniff a set of odorants systematically, has been recommended as an affordable and effective treatment for decreased olfactory function [8, 9].

OT was initially presented as a treatment for patients with olfactory loss of various causes, with exposure to four odors (rose, lemon, cloves, and eucalyptus) twice daily [10]. Over 12 weeks, the experimental group improved in olfactory performance, objectively measured by the Sniffin’ Sticks test [11]. OT has been validated and has been recommended for treating olfactory dysfunction over the last decades [9, 12–14]. For instance, patients with post-viral olfactory dysfunction demonstrated notable improvements in olfactory function, reaching a clinically significant level as assessed by the Sniffin’ Sticks test after OT [15]. Yet OT still poses some challenges such as the high rate of dropout, and different setups for the training regimen [9, 16].

So far, the majority of research focused on the improvement of olfactory function after OT in the clinic [9, 17, 18], yet there is still a lack of studies investigating OT from the patients’ point of view. It is highly important to obtain the patient’s perspective and investigate the characteristics of people who do or do not participate in OT and to learn about possible reasons for such different behaviors related to OT. In fact, patients have a high chance of exhibiting depressive symptoms when facing difficulties with adjustment to their smell loss [19].

This study aimed to investigate the opinions of people who were disturbed by their smell problems. We were specifically interested in the difference between those who participated in OT and those who did not perform OT, and especially the reasons and potential barriers to performing OT.

## Materials and methods

### Sample

Between January 2021 and January 2023, a dataset including 450 people with olfactory dysfunction was obtained via an anonymous online questionnaire, which was launched on an informational website about olfactory loss. They were divided into OT (*n* = 161), and No OT (*n* = 289) groups based on their OT participation. All participants took part in the study voluntarily and agreed with the consent. All aspects of this anonymous survey were executed according to the Declaration of Helsinki for studies on human subjects and approved by the University of Tennessee IRB review for research involving human subjects (IRB # 19-05253-XM).

### Questionnaire

The Sense of Smell Questionnaire was mainly targeted to obtain perspectives from people who were disturbed by their smell problems. It consisted of several parts, for example, the demographic information (e.g. age range and sex) and general questions such as the causes and onset of their smell loss based on their opinion. Questions were also included regarding parosmia, phantosmia, OT, and quality of life [20]. For instance, “When was the onset of your smell problem”, “Have you seen a doctor for your condition?”, “Have you seen a specialist, such as an Ear, Nose, and Throat (ENT) doctor or neurologist, for your condition?”, “Do you have parosmia (distorted sense of smell)?”, “How often are you aware of your smell problem?”, or “Do you think smell loss has led to a loss in your quality of life?”. The list of questions included in the analyses can be found in the supplementary (S1). The present study focused on the OT part of the survey and divided patients into two groups according to their responses to the question “Are you doing smell training?” According to previous research suggesting that parosmia and phantosmia exhibit unique demographic profiles, medical backgrounds, and impacts on quality of life [20], we removed individuals reporting both parosmia and phantosmia, retaining those who had either parosmia or phantosmia or neither of these conditions in the analysis. In addition, the question “How has your problem changed since it started” was considered a change in impairment (or olfactory condition) and its response (worsened/unchanged/improved) was recoded to be continuous as “-1, 0, and 1” respectively. Symptoms were calculated by counting the number of symptoms including “Stuffy nose, sneezing, facial pain, allergies, polyps, and others”.

### Data analysis

Statistical Package for the Social Sciences (SPSS, Armonk, NY, USA; version 29.0) was used for data analysis. A series of Chi-square analyses or independent t-tests were utilized to investigate the difference between groups (No OT * OT) and demographic and medical information, namely age range, gender, etiology (accident/infection/chronic rhinosinusitis), onset, symptom count, the change of olfactory condition, parosmia and phantosmia, awareness (less frequent than daily/daily/constantly) and the loss of the quality of life for their smell problems. Numeric coding was given based on the description where the higher number indicated older age, later onset, better change of olfactory condition, greater quality of life loss, and more frequent awareness of their olfactory loss, respectively. Furthermore, logistic regression analysis was utilized to determine OT participation. For those in the OT groups, we built a regression model to investigate the relationship between the duration of OT (< 4 weeks * 4–8 weeks * 8–12 weeks * 12–16 weeks * >16 weeks, coded numeric from 1 to 5) and age, gender and the loss of quality of life. For those in the No OT group, we ran Chi-square analyses to investigate the reasons for not performing OT were analyzed regarding age and gender. A two-tailed *p*-value below 0.05 denoted significance. Adjusted standardized residuals were used for chi-square post hoc analysis where an absolute residual greater than 2 indicates significance [21].

## Results

Women predominated in the sample (78%, details in Table 1). Participants were distributed in the older age range. However, neither age nor gender ratio differed in whether people participated in OT or not (both *p >* 0.7).

**Table 1:** Comparative outcomes of patients engaging in olfactory training versus non-participants

Variables	No OT (*n* = 289)			OT (*n* = 161)			χ^2^/t	*p*
	*N*	%	AR	*N*	%	AR		
***Gender***							0.00	0.99
**Men**	63	64.3	0	35	35.7	0		
**Women**	226	64.2	0	126	35.8	0		
***Etiology***								
**Accident**	19	82.3	1.9	4	17.4	-1.9	9.61	**0.008**
**Infection**	247	61.8	-3.1	153	38.3	3.1		
**Chronic rhinosinusitis**	23	85.2	2.3	4	14.8	-2.3		
***Parosmia & Phantosmia***							5.38	0.068
**Neither**	128	67.4	1.2	62	32.6	-1.2		
**Parosmia**	120	58.8	-2.2	84	41.2	2.2		
**Phantosmia**	41	73.2	1.5	15	26.8	-1.5		
***Age (mean ± SD)***	3.95 ± 1.64			3.90 ± 1.43			0.33	0.71
***Onset***	2.34 ± 1.21			1.99 ± 0.95			3.13	**0.002**
***Olfactory condition***	0.07 ± 0.76			0.24 ± 0.84			-2.05	**0.041**
***Loss of QoL***	2.51 ± 1.15			3.06 ± 1.02			-5.08	**< 0.001**
***Awareness***	4.52 ± 0.81			4.75 ± 0.45			-3.31	**0.002**
***Symptom count***	0.98 ± 1.19			0.93 ± 1.19			1.34	0.18

Note: OT = Olfactory training, AR = adjusted residuals, QoL = Quality of life

### Factors predicting OT participation

**Fig. 1:**
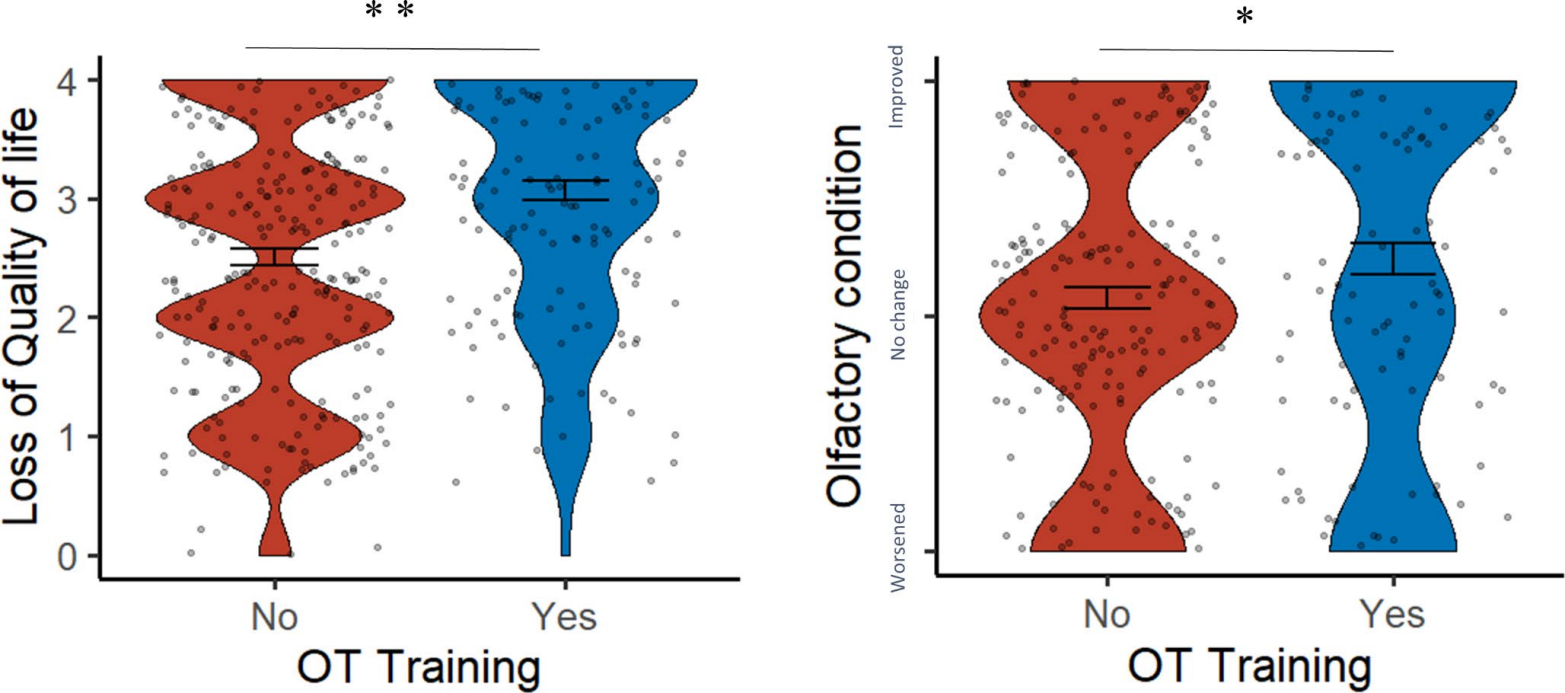
Violin plots for quality of life loss and olfactory condition between the groups of olfactory training (OT) and no olfactory training. *p < 0.05, **p < 0.01

Binary regression analysis revealed that the degree of quality of life loss and olfactory condition contributed significantly to predicting OT participation (Omnibus *χ*^*2*^ = 46.36, *p* < 0.001; Table 2). Patients with greater loss of quality of life caused by their smell problems (Fig. 1A, *p* < 0.001) and who considered their olfactory condition improved were more likely to participate in OT (Fig. 1B, *p* < 0.001).

**Table 2:** Binary regression analysis: factors influencing participation in olfactory training

Variables	B	S.E.	Wald	df	Sig.	Exp(B)	95% C.I.	
							Lower	Upper
***Etiology (ref: Accident)***			1.11	2	0.57			
**Infection**	0.53	0.64	0.70	1	0.40	1.71	0.49	5.94
**Chronic rhinosinusitis**	0.02	0.82	0	1	0.98	1.02	0.20	5.12
***Parosmia & Phantosmia*** ***(ref: Neither)***			4.22	2	0.12			
**Parosmia**	0.48	0.25	3.65	1	0.06	1.61	0.99	2.624
**Phantosmia**	-0.04	0.39	0.01	1	0.92	0.96	0.45	2.068
**Onset**	-0.12	0.13	0.95	1	0.33	0.88	0.69	1.13
**Awareness**	0.15	0.23	0.43	1	0.51	1.16	0.74	1.81
**Symptoms count**	-0.02	0.11	0.04	1	0.84	0.98	0.80	1.20
**Quality of life loss**	0.52	0.12	19.92	1	**< 0.001**	1.69	1.34	2.12
**Olfactory condition**	0.42	0.15	8.18	1	**< 0.001**	1.53	1.14	2.04
**Constant**	-3.25	1.20	7.31	1	0.01	0.04		

The causes of smell problems differed between groups (No OT vs. OT; *χ*^*2*^ = 9.61, *p* = 0.008). Post hoc analysis showed that participants with post-infectious olfactory loss tended to conduct OT (89%, adjusted residuals = 3.1), while patients with chronic rhinosinusitis were not engaged in OT participation (adjusted residuals = 2.3). Patients with chronic rhinosinusitis are often congested via mucus or polyps and tend to have more stuffy or blocked nose, leading to a barrier to participate in a smelling activity. We further checked this result by conducting a one-way ANOVA (accident/infection/chronic rhinosinusitis) and found that patients with chronic rhinosinusitis had higher symptom counts than both of the other groups (both *p* < 0.001). Parosmia was a common complaint in the present study (45%), but did not differ between groups (No OT vs. OT, *χ*^*2*^ = 5.38, *p* = 0.068), yet post hoc analysis suggested patients with solely parosmia were more likely to participate in OT (adjusted residuals = 2.2). Patients who participated in OT had a later onset of olfactory loss (*t* = 3.13, *p* = 0.002), greater loss of quality of life (*t* = 5.08, *p* < 0.001), improved olfactory condition (*t* = 2.05, *p* = 0.041), and more awareness of their condition (*t* = 3.31, *p* = 0.001).

### OT duration

**Fig. 2:**
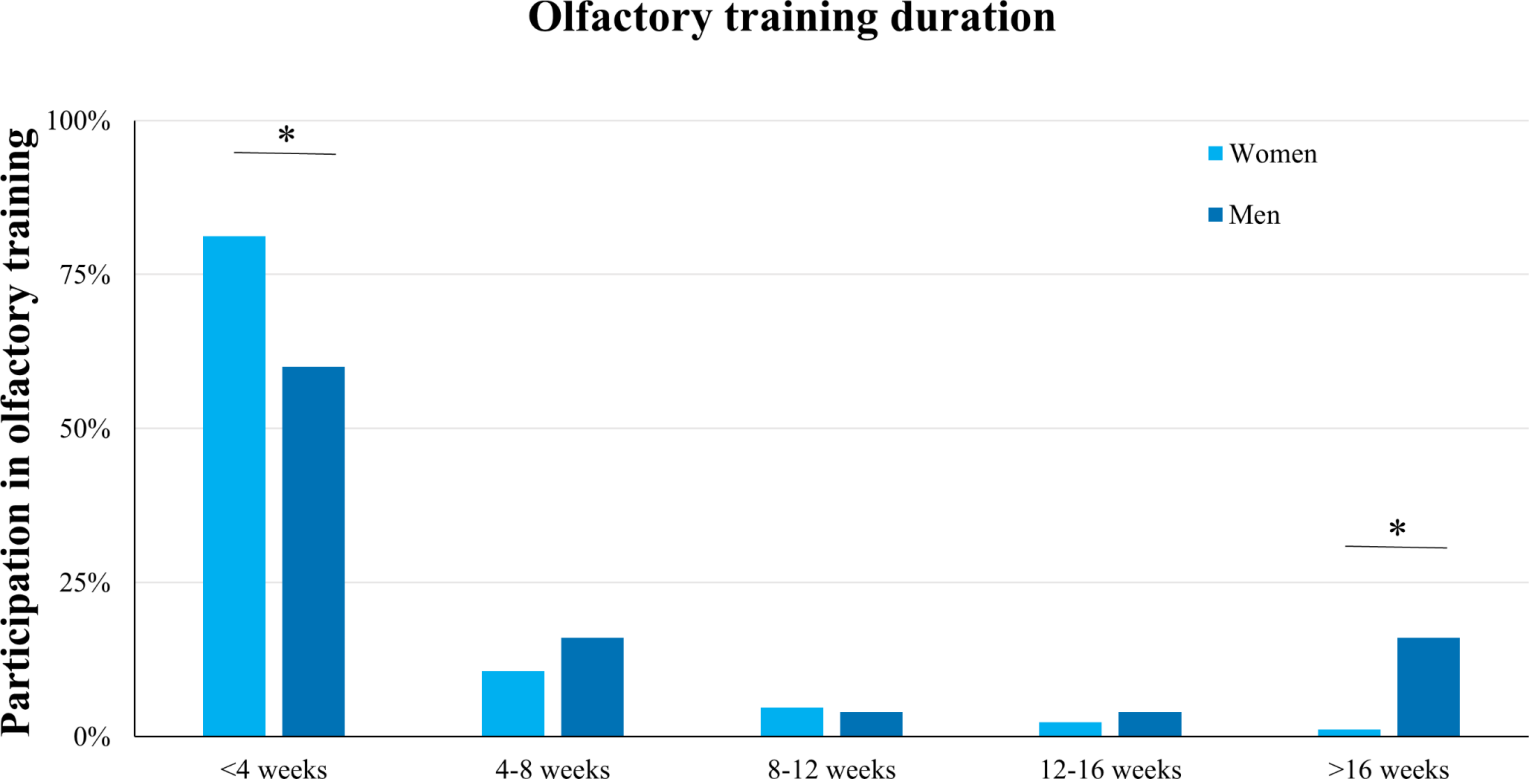
The distribution of olfactory training duration in patients who participated in olfactory training. The majority of patients performed less than 4 weeks and women tended to get involved in olfactory training in a shorter duration compared to men

The majority of participants performed OT for less than four weeks (76.4%, Fig. 2). Regression analyses showed that gender was associated with OT duration (F = 4.38, *p* = 0.006), whereas men were more likely to have a longer OT duration (*p* = 0.002). No other significant results were found (all *p* > 0.05).

### Reasons for not performing OT

**Fig. 3:**
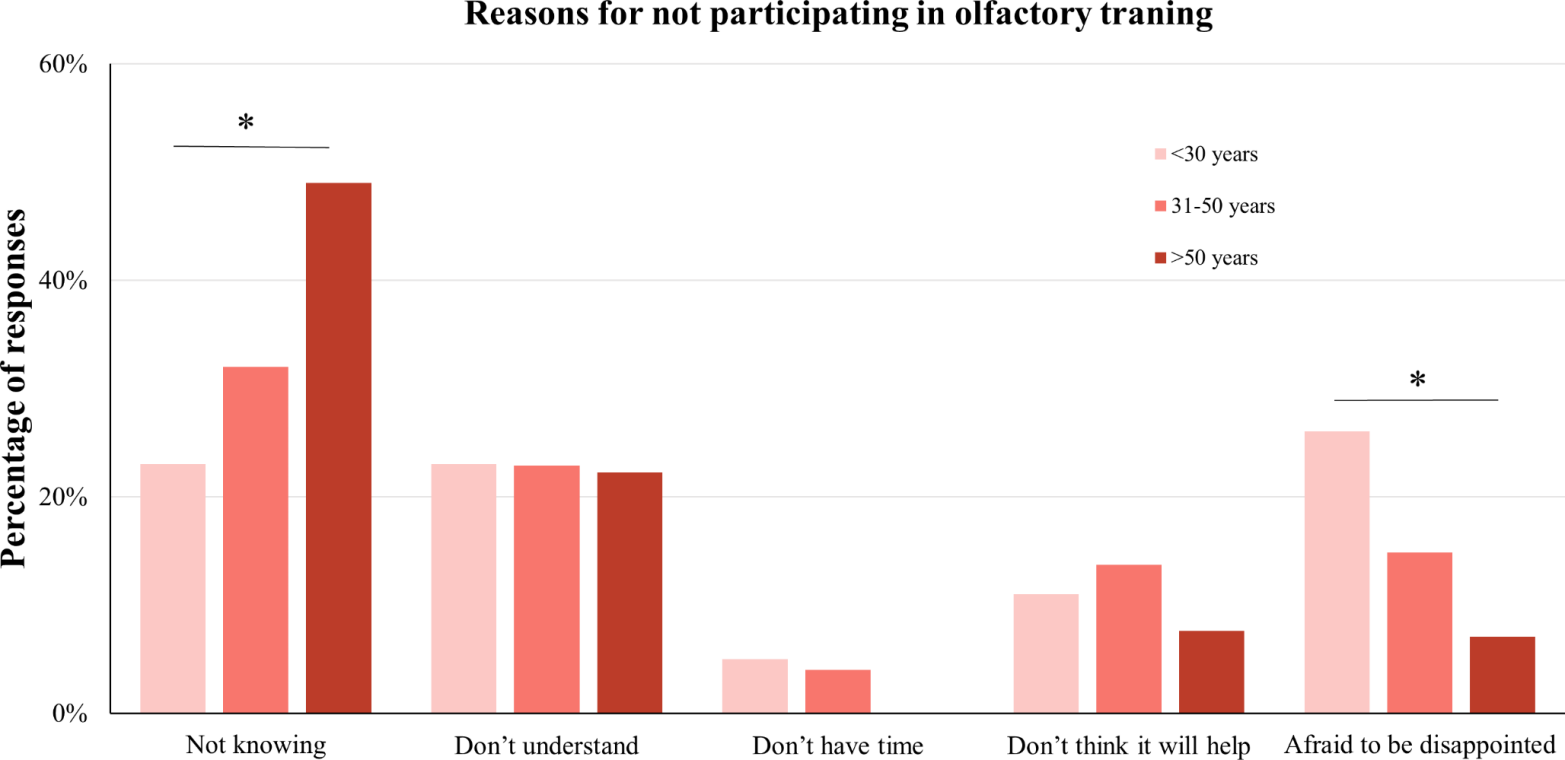
The distribution of reasons for non-participation in olfactory training among patients differed by age. The general major reason is unaware of this method, and this is especially for patients older than 50 years. Patients under 30 years old were afraid to be disappointed by olfactory training

In the No OT group, the most common reason for not conducting OT was not knowing about OT (37%, Fig. 3). To explore the reasons behind the lack of awareness about OT, we conducted further analysis by examining the proportion of patients who sought medical advice from doctors or ENT specialists (refer to S2). A third of the NO OT patients (37.7%, *n* = 109) sought consultation from a doctor regarding their condition. In comparison, a larger percentage of patients who did participate in OT consulted their doctor (54%, *N* = 87), but no differences were seen between NO OT and OT groups who consulted an ENT specialist (65.1% and 65.5%, respectively). However, regardless of seeing a general practitioner or ENT specialist, half of NO OT patients chose “did not know” about OT as a leading reason they did not participate [51% (*N* = 45) and 50% (*N* = 28), refer to S3]. This indicates that a significant portion of patients who sought medical advice, including specialized care, remained uninformed about OT. Gender had no association with the reason for not performing OT (*p* = 0.95). Interestingly, people over 50 years were more likely to be unaware of the method of OT compared with the younger population aged below 30 years (*χ*^*2*^ = 43.86, *p* < 0.001; adjusted residuals = 4.5), whereas a higher proportion (26%) of those aged below 30 were more afraid to be disappointed by OT (adjusted residuals = 3.9).

## Discussion

The primary objective of this study was to investigate the perspectives of people with smell problems concerning OT participation. The main outcomes were: (i) The greater loss of quality of life and improved olfactory condition determined the OT participation; (ii) Limited training sessions were predominant in participants conducting OT, while reasons for not participating in OT differed by age.

Lower quality of life due to smell loss encouraged patients to pursue the treatment of OT. In fact, smell loss disturbs people’s quality of life through altered food enjoyment, social relationships, and even working life, heightening the chance of being depressive [2, 22]. In line with previous studies, patients who experience a significant decline in their quality of life in the present study were more likely to seek assistance, such as through OT. Olfactory condition also contributed to OT in the present study, where those who had improved condition were involved actively in OT. This association is consistent with previous studies suggesting the improvement of olfactory function after OT [9, 17, 23, 24].

OT participation is relevant to other factors, such as etiology, which could be related to various training efficacies in improving olfactory function for different etiologies [9]. Additional analyses including patients with only infectious reasons and the main results in this subgroup were unchanged. Further analysis suggested that the inactive OT involvement in patients with chronic rhinosinusitis could be on account of more symptoms, for instance, the stuffy nose. We found that patients who participated in OT had a relatively shorter onset of olfactory loss. One possible explanation would be that those who had an early onset could, to some extent, adjust to their smell problem, thus they were probably less motivated to pursue treatment. Parosmia is a common complaint [20, 25], which in most cases links to unpleasant odorous sensations leading to a substantial decline in one’s quality of life [20, 26]. People with parosmia may find themselves deeply troubled by the distressing olfactory distortion. In turn, this may lead to a greater inclination to consider and pursue OT as a recommended treatment. This observation may also extend to those who exhibited more awareness and the improvement of their smell problems, subsequently motivating their active engagement in OT.

The present study found that the majority of patients performed OT for less than a month. Generally, the duration of OT is recommended at least three months and longer, and studies have shown that the longer duration provides more improvement in olfactory function [17, 27]. In the clinical context, one month of training may not be sufficient to produce significant improvement [28], whereas prolonged OT relates to a higher chance of improvement [29–31]. Interestingly, men reported that they spent more time with OT than women in the present study, while previous research indicated that gender had no strong association with olfactory recovery after OT [32, 33]. Overall, it seems advisable for clinicians to instruct patients on the importance of adhering to OT for a “prescribed” period.

As for the reasons for not conducting OT, we found that the factors for limited engagement in OT were associated with age. OT is still widely unknown, with more than one third of patients never heard of it, even though a large number of patients visited general practitioners or even ENT-related specialists for their smell problems already. This was especially true for the older population. The post hoc analysis utilizing adjusted residuals from the Chi-square analysis suggested that the majority of older people considered OT as not time-consuming, that it would be helpful, and that they were not afraid to be disappointed. On the other hand, younger participants were more cautious about the training effect. In the practice of dealing with patients with smell problems, we anecdotally observed that younger individuals who are active on social media platforms may be misled by negative encounters with short-duration OT treatments shared online. These experiences can contribute to a misconception that OT lacks effectiveness, as users may not have experienced its full benefits due to their abbreviated treatment duration. One crucial insight for caregivers derived from these findings is the need to raise awareness about OT and promote the basic knowledge of how and why OT works, and what are its benefits, with the adherence to OT for 3 months or longer.

The present findings should be interpreted in light of certain limitations. Firstly, the study adopted a cross-sectional design that can only show associations between variables. Additionally, the utilization of an online survey introduces a significant volunteer bias, as participation was voluntary, which may affect the representativeness of the sample and limit the generalizability of the findings.

## Conclusion

In conclusion, based on results from a survey, patients who perceived a substantial negative impact on their quality of life due to their smell problems and who considered their olfactory condition improved were more inclined to engage in OT. Furthermore, it is noteworthy that a significant portion of patients did not adhere to the recommended training regimen for an adequate duration. Common barriers to OT participation included a lack of awareness regarding the availability of OT among older people and a lack of confidence in the effectiveness of OT in the younger population. Recommendations for clinicians, based on the findings of this study together with the fruitful findings of OT effectiveness during the last decades, include actively advocating OT to patients while emphasizing the critical significance of adhering to the suggested training regimen.

## Electronic supplementary material

Below is the link to the electronic supplementary material.

Supplementary Material 1
