# Supplementary material for: Olfactory training: perspective from people who were disturbed by their smell problems
Source: Eur Arch Otorhinolaryngol. 2024 Aug 23;281(12):6423–30. doi: 10.1007/s00405-024-08911-7 (PMC11564411; doi:10.1007/s00405-024-08911-7)
Supplement: Supplementary file 1 — Supplementary Material 1 [file 405_2024_8911_MOESM1_ESM.docx]

S1 Items in the Sense of Smell Questionnaire

| Variable | Question | Levels |
| --- | --- | --- |
| Age | Your Age | 21-30, 31-40, 41-50, 51-60, 61-70, 71 and over |
| Gender | Your Gender | Female, Male, Other, Prefer not to say |
| Onset | When was the onset of your smell/taste problem? | less_than_3_months_ago, 3_12_months_ago, 1_2_years_ago, more_than_2_years_ago, as_long_as_i_can_remember, i_dont_know |
| Olfactory condition | How has your problem changed since it started? | i_dont_know_if_it_has_changed_since_it_started, the_situation_has_worsened, the_situation_is_unchanged, there_has_been_improvement |
| Cause | In your opinion, what might have been the cause? | accident, cold_infection, dry_mouth, medication, nasal_polyps_chronic_rhinosinusitis, other, surgery |
| Symptoms | Have any of these symptoms? | CATA: Stuffy nose, Sneezing, Allergies, Polyps, Facial pain, Other |
| Doctor visit | Have you seen a doctor for your condition? | no, yes |
| Specialist visit | Have you seen a specialist, such as an Ear, Nose and Throat (ENT) doctor or neurologist, for your condition? | no, yes |
| Parosmia | Do you have parosmia (distorted sense of smell)? | no, yes |
| Phantosmia | Do you experience smells that are not present (phantosmia)? | no, yes |
| Olfactory Training | Are you doing smell training? | yes; no, I have tried smell training in the past but stopped; no, I have never tried smell training |
| Olfactory Training never | If you have not tried smell training, what is the reason? | I do not think it would help me, I am afraid to start, because I might be disappointed if it doesn’t work, I don’t understand it, I don’t have time, Other |
| Awareness | How often are you aware of your smell problem? | constantly, daily, weekly, monthly, it_doesnt_bother_me |
| Quality of life | Do you think smell loss has led to a loss in your quality of life? | greatly, considerably, moderately, slightly, not_at_all |

**S2** Percentage of responses to the questions “Have you seen a doctor for your condition?” and “Have you seen a specialist, such as an Ear, Nose, and Throat (ENT) doctor or neurologist, for your condition?” in the whole sample.

|  |  | No OT | | OT | | Total | |
| --- | --- | --- | --- | --- | --- | --- | --- |
|  |  | N | % | N | % | N | % |
| Have you seen a doctor for your condition? | no | 180 | 62.3% | 74 | 46.0% | 254 | 56.4% |
|  | yes | 109 | 37.7% | 87 | 54.0% | 196 | 43.6% |
| Total |  | 289 | 100.0% | 161 | 100.0% | 450 | 100.0% |
| Have you seen a specialist, such as an Ear, Nose, and Throat (ENT) doctor or neurologist, for your condition? | no | 38 | 34.9% | 30 | 34.5% | 68 | 34.7% |
|  | yes | 71 | 65.1% | 57 | 65.5% | 128 | 65.3% |
| Total |  | 109 | 100.0% | 87 | 100.0% | 196 | 100.0% |

**S3** Percentage of responses to the questions “Have you seen a doctor for your condition?” and “Have you seen a specialist, such as an Ear, Nose, and Throat (ENT) doctor or neurologist, for your condition?” in no olfactory training group.

|  | | Have you seen a doctor for your condition? | | | | | |
| --- | --- | --- | --- | --- | --- | --- | --- |
|  | | no | | yes | | Total | |
|  |  | N | % | N | % | N | % |
| If you have not tried smell training, what is the reason? | Not knowing | 60 | 37.7% | 45 | 51.1% | 105 | 42.5% |
|  | Don’t understand | 47 | 29.6% | 14 | 15.9% | 61 | 24.7% |
|  | Don’t have time | 7 | 4.4% | 2 | 2.3% | 9 | 3.6% |
|  | Don’t think it will help | 12 | 7.5% | 14 | 15.9% | 26 | 10.5% |
|  | Afraid to be disappointed | 33 | 20.8% | 13 | 14.8% | 46 | 18.6% |
| Total | | 159 | 100.0% | 88 | 100.0% | 247 | 100.0% |

|  | | Have you seen a specialist, such as an Ear, Nose, and Throat (ENT) doctor or neurologist, for your condition? | | | | | |
| --- | --- | --- | --- | --- | --- | --- | --- |
|  | | no | | yes | | Total | |
|  |  | N | % | N | % | N | % |
| If you have not tried smell training, what is the reason? | Not knowing | 17 | 53.1% | 28 | 50.0% | 45 | 51.1% |
|  | Don’t understand | 5 | 15.6% | 9 | 16.1% | 14 | 15.9% |
|  | Don’t have time | 1 | 3.1% | 1 | 1.8% | 2 | 2.3% |
|  | Don’t think it will help | 4 | 12.5% | 10 | 17.9% | 14 | 15.9% |
|  | Afraid to be disappointed | 5 | 15.6% | 8 | 14.3% | 13 | 14.8% |
| Total | | 32 | 100.0% | 56 | 100.0% | 88 | 100.0% |
